# 

**Published:** 1883-04

**Authors:** 


PAGE

ORIGINAL COMMUNICATIONS.—Acute Parenchymatous Degeneration of the Kidneys. William Henry Porter, M.D. ........ 87
The Extent to which Animal Forms Undrego Change. C. M. O'Leary, M.D., LL.D ........ 94
Some Recent Contributions to the Physiology of the Nervous System of our Domestic Animals. Charles L. Dana, A.M., M.D ........ 101
Are there Physiological Affinities in the Blood of Each Breed of Animals. A. S. Heath, M.D... 104
Abortion in Cows. Ehrick Parmly, M.D........ 109
Atavism. Hubbard W. Mitchell, M.D ........ 113
Milk Sickness. N. S. Townsend, M.D........ 117
The Teeth of the horse and their Diseases. R. Jennings, V.S ........ 121
Oleum Gaultheria—Oil of Wintergreen. E. S. Bates, M.D........ 128
Inoculation of Animals for the Prevention of Pleuro-Pneumonia ........ 130
EDITORIAL.—The Progress of Comparative Pathology as regards Infectious and other Diseases—Abortion in Cows—One-Stool Veterinary Colleges—A Western Veterinary Journal—To the Veterinary Profession at Large—Journalistic Changes........ 132–141
REPORT OF COMMENCEMENTS AND SOCIETIES........ 142
CASE DEPARTMENT—Lesions in a Case of Immobilite—Traumatic or Idiopathic Tetanus—Idiopathic Tetanus—Acute Parenchymatous Nephritis—Acute Parenchymatous Degeneration of the Kidneys in a Mare—Recurring Cystic Tumors of the Supra-Scapular Region—Penetrating Wound of the Abdomen—Intestinal Calculi Lodged in Diaphragmatic Curvature of the Colon—Hæmaturia Associated with Nephritis—Fracture of the Internal Metatarsal, or Internal Splint Bone—Chronic Rheumatic Arthritis with Osteophatic Outgrowths—Fracture of the Os Inominata........ 150–164
REVIEWS.—Veterinary Medicines—Report of the Commissioners of Agriculture—Books end Pamphlets Received........ 165–166
OBITUARY ........ 166
REPORTS OF MONTREAL VETERINARY COLLEGE........ 167
TABLES IN COMPARATIVE PHYSIOLOGY........ 170
PROGRESS OF VETERINARY SCIENCE.—Ascarias Mystax in Cats—Inoculation of Monkeys with Syphilis—Sheep Fluke—Etiology of Glanders—Swine Plague—Influence of Digitaline in the Heart of the Terrapin—Fibro-Myomata of the Uterus of the Sea-Lion—Test for Idoform—Tinctura Idoformi Composita—Male Fern—Osseous Deformities in the Lower Animals—Diptheria in the Bladder—Equine Scarlatinal Virus—Intestinal Obstruction—Pasteur and Koch—The Newly Discovered Prophylactic Vaccine for Rouget—Experiments on Animals with Male Fern—Movements of Bowels in Health and Disease—Medicine as Practised by Animals—Form of the Pulse Wave—The Prevention of Hydrophobia—Pasteur on Rabies—The Germ of Hog Cholera........ 171–179
NEWS AND MISCELLANY.—New Veterinary Schools—Number of Veterinary Surgeons in U. S.—Bitten by a Squirrel—Cold Kills Trachinæ—Contrivance to Prevent Balling of Horses' Feet—Small-Pox in Birds—Phthisis Conveyed from Dogs to Man—Loss of Cud—Ontario Veterinary Society—Report of the Treasury Cattle Commission—Hospital for Animals—Tables in Comparative Physiology—The Vivisection Question—A Horse Calls on a Doctor—Intelligence in Animals—A Case of Filaria Oculi Occurring in Both Eyes of a Horse—Bacteria—Parasites in American Pork—Presentation—Prof. Virchow's Health—Red Water in Cattle—Abnormal Periods of Gestation........ 180–184

